# Polygenic Risk Score and Risk Factors for Preeclampsia and Gestational Hypertension

**DOI:** 10.3390/jpm12111826

**Published:** 2022-11-03

**Authors:** Marija Majda Perišić, Klemo Vladimir, Sarah Karpov, Mario Štorga, Ali Mostashari, Raya Khanin

**Affiliations:** 1 LifeNome Inc., New York, NY 10018, USA; 2 Faculty of Mechanical Engineering and Naval Architecture, University of Zagreb, 10000 Zagreb, Croatia; 3 Faculty of Electrical Engineering and Computing, University of Zagreb, 10000 Zagreb, Croatia; 4 Bioinformatics Core, Memorial Sloan-Kettering Cancer Center, New York, NY 10065, USA

**Keywords:** preeclampsia, gestational hypertension, eclampsia, pregnancy, polygenic risk score, gwas, machine learning

## Abstract

Preeclampsia and gestational hypertensive disorders (GHD) are common complications of pregnancy that adversely affect maternal and offspring health, often with long-term consequences. High BMI, advanced age, and pre-existing conditions are known risk factors for GHD. Yet, assessing a woman’s risk of GHD based on only these characteristics needs to be reevaluated in order to identify at-risk women, facilitate early diagnosis, and implement lifestyle recommendations. This study demonstrates that a risk score developed with machine learning from the case-control genetics dataset can be used as an early screening test for GHD. We further confirm BMI as a risk factor for GHD and investigate a relationship between GHD and genetically constructed anthropometric measures and biomarkers. Our results show that polygenic risk score can be used as an early screening tool that, together with other known risk factors and medical history, would assist in identifying women at higher risk of GHD before its onset to enable stratification of patients into low-risk and high-risk groups for monitoring and preventative programs to mitigate the risks.

## 1. Introduction

Hypertensive disorders of pregnancy or gestational hypertensive disorders (GHD) are the most common pregnancy complications. In fact, GHD constitute the leading causes of maternal and perinatal morbidity and even mortality. About one-quarter of all global maternal deaths are due to GHD [1]. The lack of precision in diagnosing GHD makes it difficult to estimate global prevalence accurately, but recent reports have pointed to an increase in the prevalence of GHD, specifically preeclampsia. In the United States alone, there has been a 25% increase in the rate of preeclampsia from the years 1987–2004 [2]. It is estimated to complicate 2–8% of pregnancies worldwide, making it one of the leading causes of maternal morbidity alongside other GHD [3].

Preeclampsia is categorized by sustained high maternal blood pressure (hypertension) that must be identified after 20 weeks of gestation [3], and often requires early delivery of the infant. If the condition worsens, maternal hypertension may cause seizures, brain bleeding (hemorrhagic stroke), coma, or hypertensive encephalopathy. Severe preeclampsia can also cause fetal impairment of blood and oxygen flow, which often results in growth complications or even stillbirth.

The standard screening test for GHD has always been measuring one’s blood pressure, which is usually only useful when the disease has already started to progress. In this case, risk factors become imperative in assessing one’s chances of developing GHD. Women with health conditions such as high BMI, adiposity, hypertension, heart disease, diabetes, or kidney disease before they become pregnant have an increased risk of developing preeclampsia [4,5]. Additional moderate risk factors for developing GHD include advanced maternal age and family history of preeclampsia.

Although these criteria are easy to apply, they perform with poor sensitivity [6]. Prediction performance for late gestation preeclampsia is even less accurate, further demonstrating the inefficient nature of these diagnostic criteria. More accurate clinical tests exist to predict preterm GHD or preeclampsia, but due to the high costs of implementation, universal access is hard to implement [1,7]. A significant unmet need remains for predictive screening tests to reduce the disease burden associated with late-onset, severe GHD.

Large-scale genetic studies estimated heritability for GHD, or more specifically preeclampsia, to be around 55% [8]. An analysis of over 70,100 pregnancies from Swedish families, that included data from 244,564 siblings, reported that 35% of the variance in occurrences of preeclampsia was attributable to maternal genetics, 20% to fetal genetics (with a similar contribution of maternal and paternal genetics), 13% to the couple effect, less than 1% to shared sibling environment, and 32% to other unmeasured factors [8].

Furthermore, a number of gene candidate-based studies of preeclampsia were conducted, resulting in a list of genes associated with the risk [9]. Among the genes reported to be associated with preeclampsia are MTHFR (rs1801133), F5 (rs6025) [10], NOS3 [11], PPAR*γ* [12], ERAP2 [13], STOX1, CORIN, EPHX1, INO80B [14], GSTP1 [15], FGF5 [16], a role for the X chromosome [17] and several other chromosomal regions. According to this set of studies, some genes were found to affect susceptibility to preeclampsia in specific populations [18,19].

Each study started with a hypothesis on the molecular mechanisms underlying preeclampsia and analyzed a few genes. Since preeclampsia, and other GHD, are complex diseases, it is imperative to use large-scale genomics data to identify genetic variations and genes associated with the risk of GHD. Hence, the current study aims to develop a predictive polygenic risk score (PGS) for GHD that can be integrated into clinical practice. We, therefore, utilize genetics data from the UK Biobank (UKBB) for the development and validation of a PGS.

## 2. Materials and Methods

### 2.1. Participants

This study utilizes the data of UKBB (www.ukbiobank.ac.uk) (accessed on 5 March 2022), which is a prospective cohort of 502,637 people aged between 37 and 73 and recruited from 2006 to 2010 from across the UK. The participants’ medical, socio-demographic, lifestyle, environmental, and genetic information was collected via detailed questionnaires and clinical assessment and linked with hospital admission and mortality data. The analysis reported in this paper included 273,309 UKBB participants self-identifying as females, for which no mismatch between self-reported and genetic gender was detected.

All procedures and data collection in UKBB were approved by the UKBB Research Ethics Committee (reference number 11/NW/0274), with participants providing full written informed consent for participation in UKBB and subsequent use of their data for approved applications.

### 2.2. Gestational Hypertension/Preeclampsia Cases

Data on gestational hypertension was retrieved from self-reported data on gestational hypertension/preeclampsia Data-Field 20002, data field 1073 (2008 cases). We further added hospital in-patient episode data with diagnosis codes O13 “Gestational [pregnancy-induced] hypertension without significant proteinuria” (581 cases) and O14 Gestational [pregnancy-induced] hypertension with significant proteinuria (318 cases) (“Diagnoses—ICD10”). Case definitions for preeclampsia included eclampsia cases to capture cases in which the former may have developed into the latter. For more focused study on preeclampsia/gestational hypertension patients with diagnosis code O13 were not included in the cases group.

The control group contains women who were pregnant and gave live births but did not report gestational diabetes, or gestational hypertension/preeclampsia, and recurrent pregnancy losses. Furthermore, women with preexisting conditions were excluded from the control group. Specifically, for the control pool we used data for women who had at least one live birth without complications for gestational diabetes or gestational hypertension/preeclampsia, or eclampsia, or preexisting diabetes. Specifically, we included women with the codes related to live birth and pregnancy O2–O9 or Z34.8, Z37.0, Z37.2, Z37.3, Z37.5, Z37.6, Z38.1, Z38.3, Z38.6, Z39; but excluding those related to codes relevant for gestational diabetes, gestational hypertension, eclampsia (codes O10–O16), and pre-existing diabetes (O24.0, O24.1, O24.2, O24.3, O24.9). For a more general control population group, these cases were not exluded.

### 2.3. Genotype and Phenotype Data

To identify variants for building the PGS we utilized the results from Neale lab GWAS of UKBB phenotypes www.nealelab.is/uk-biobank/ (accessed on 9 June 2022). We combined the results from traits related to gestational hypertension/preeclampsia self-report diagnoses (field 20002, code 1073) and doctor-diagnosed gestational hypertension (field 41270, codes O13 and O14). We selected SNPs below the significance cutoff $1\times 10^{-5}$. Overall, this analysis yielded 329 distinct SNPs. The list of relevant SNPs was further extended based on studies [20,21] and the GeneAtlas (http://geneatlas.roslin.ed.ac.uk accessed on 20 June 2022) below the significant cutoff $1\times 10^{-5}$. Two SNPs rs113935429 and rs201454025 (identified as relevant in [20]) and rs146665468 (from the GeneAtlas) were not found in the UKBB dataset and hence were excluded from further analysis. Overall, the procedure resulted in a final set of 375 SNPs that were further considered in the analysis.

To consider the relationship between the genetic risk of GHD and BMI, we analyzed data on the participants’ body mass index (BMI). In instances where the participants’ BMI (data-field 21001) was recorded repeatedly, the latest BMI reported was taken as a BMI estimate. If an individuals’ BMI was not reported or below 18.5 they were omitted from this analysis.

### 2.4. Procedure for Learning the Polygenic Risk Scores

The full procedure for learning the Polygenic Risk Score was described in detail in [22]. Briefly, we use a machine learning procedure to build a PGS as a risk-weighted sum of the genetic variants, where the number of effect alleles is represented by either 0, 1, or 2. The starting set of SNPs (features) were first clumped using PLINK’s LD-based clumping procedure resulting in 115 unique SNPs used in further modeling. The number of cases and controls was balanced by randomly sampling the full set of controls (10 times) so that the number of controls is 4-times bigger than the number of cases. Each set of cases and controls was then used in a logistics regression model by repeating the 10-fold cross-validation process ten times. Once the ten models were trained, the best model was selected based on the area under the receiver operating characteristic curve (AUC). 95% confidence intervals (CI)s for odds ratios (OR) were calculated as Wald intervals (or Normal approximation intervals) using the oddsratio function from the epitools package in R. Furthermore, a step-wise learning procedure based on a forward-selection method and the Akaike Information Criterion was also performed using the stepAIC function in MASS and car R packages.

### 2.5. Mendelian Randomization

TwoSampleMR in R package was utilized for performing Mendelian Randomization analyses. The significance threshold for genetic instruments is $5\times 10^{-8}$. Pleiotropy was evaluated based on the intercept calculated by MR-Egger regression using mr_pleiotropy_test with *p*-value threshold p=0.05. Exposure-outcome relationships that change by at least 10% in the odds ratio (OR≥1.1 or OR≤0.9) are reported.

### 2.6. SNP Annotation

SNPs are annotated with genes and genome-wide association studies (GWAS) using SNPnexus, a web-based variant annotation tool [23,24]. Functional analysis on gene level is performed using Functional Mapping and Annotation of Genome-Wide Association Studies, FUMA [25].

## 3. Results

### 3.1. Polygenic Risk Score

A dataset of 2787 cases and 13,400 controls for GHD from the UKBB is utilized for a case-control retrospective study. To develop a polygenic risk score for gestational hypertensive disorders, we consider those women who either self-reported gestational hypertension/preeclampsia or were diagnosed with gestational hypertension with or without proteinuria (see Methods for a detailed explanation of the selection of cases and control groups).

PGS was calculated as a weighted sum of the 115 genetic variants, which were derived by pruning from the initial set of 375 genetic variants based on Neale Lab GWAS of UKBB phenotypes (http://www.nealelab.is/uk-biobank/ and the GeneAtlas (http://geneatlas.roslin.ed.ac.uk/ (accessed on 12 October 2022) below the significance cutoff $p<10^{-5}$, and several earlier studies [20,21]. Weights for each variant were learned by utilizing a generalized linear model and logistic regression with added collinearity analysis for the predictor variables, where the best-performing model was selected based on the estimated AUC (for details, see [22]). The resulting PGS has 115 SNPs and AUC = 0.64 (Table 1 and Supplementary Table S1). We also used the stepwise procedure that resulted in 84 SNPs (Supplementary Table S1) and a slightly lower AUC = 0.62.

**Figure 1:**
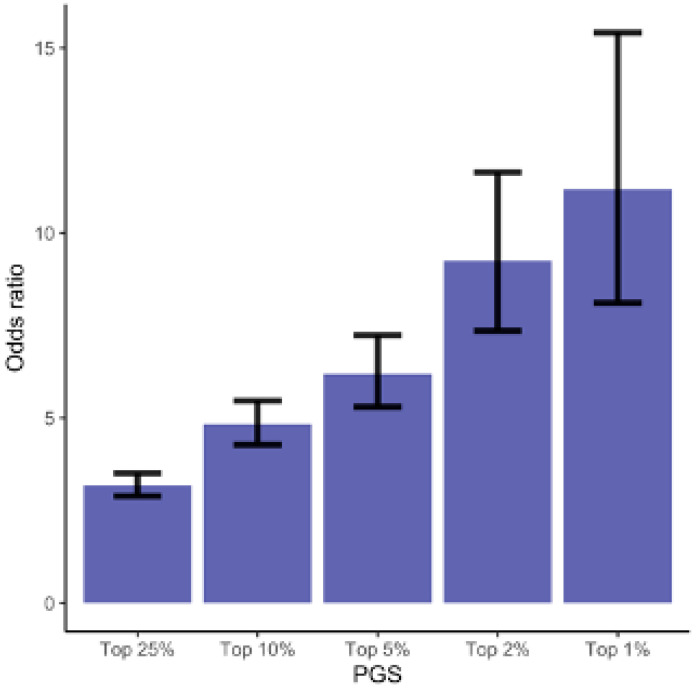
Odds Ratios for the GHD. The odds of being diagnosed with GHD for women ranked in the top 1%, 2%, 5%, 10%, and 25% of the PGS compared to the odds of developing GHD in the lower 50% of the PGS (Supplementary Table S2).

To identify at-risk group of GHD, we computed odds ratios (ORs) for GHD by comparing the women ranked in the top 1%, 2%, 5%, 10%, and 25% PGS values to the women whose PGS values are in the lower 50%. Compared to women in the lowest half of the PGS, the odds ratios (ORs) for GHD are OR = 3.18 (95% CI = [2.89–3.51]) for the top quantile, OR = 4.84 (95% CI = [4.28–5.46]) for the top 10%, OR=6.19 (95% CI = [5.30–7.24] top 5%, OR = 9.25 (95% CI = [7.35–11.64] top 2%, and OR = 11.18 (5% CI = [8.11–15.41]) for the top 1% (Figure 1 and Supplementary Table S2).

To make sure our model is encompassing to the choice of controls and cases, we ran additional analyses. First, we modified the control group to be more representative of the general population. The modified controls group contains women who gave live births, including women with pre-existing conditions. This results in a slightly larger size of the control group (14,419 genotypes).The updated PGS is based on the same set of SNPs, and the changes in ORs for the top risk percentiles are minor (Supplementary Tables S1 and S2).

We further performed a more focused analysis on the preeclampsia trait defined as “new-onset hypertension and proteinuria” by removing 581 cases with diagnosis code O13 “Gestational [pregnancy-induced] hypertension without significant proteinuria”. The resulting PGS contains 101 SNPs out of 115 SNPs from the original machine learning procedure (Supplementary Table S4). Compared to women in the lowest half of the PGS, the ORs for preeclampsia are even higher: OR = 5.67 (95% CI = [4.98–6.45]) for the top 10%, OR = 7.84 (95% CI = [6.66–9.23] top 5%, and OR = 12.76 (5% CI = [9.19–17.71]) for the top 1% (Supplementary Table S4).

Therefore, the developed PGS can be utilized as an early screening tool to identify women at high risk of GHD and stratification of population into risk groups. The tool can be used pre-conception allowing for early lifestyle modifications and close monitoring during early stages of pregnancy.

### 3.2. GHD Risk and BMI

We further investigate the effect of BMI on the risk of GHD. An earlier meta-analysis of 16 studies that included a total number of 5946 samples revealed a significant relationship between BMI and the risk of preeclampsia [26]. Another case-control study [27] found that patients with a pre-pregnancy BMI of more than 30 kg/m^2^ had a nearly 4.5-fold risk of developing gestational hypertension compared to pregnant women whose pre-pregnancy BMIs were in the normal range.

**Figure 2:**
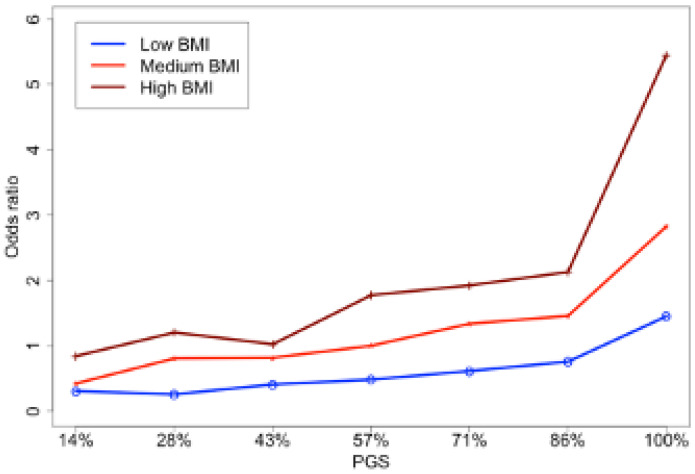
The odds ratios for GHD based on PGS for three BMI groups. The group of participants with medium-level PGS (43–57%) and medium-level BMI (25–30) is taken as a reference group.

To further elucidate the relationship between BMI and PGS for GHD, we divided all samples into three groups according to BMI: low (18.5–25), medium (25–30), and high ≥30 [28]. Additionally, the samples were divided into seven levels (i.e., septiles) according to the values of PGS This way, all samples were grouped into 21 categories based on their PGS and BMI. The odds ratios (ORs) of GHD in each group were computed relative to the group with the medium BMI and median PGS (Figure 2 and Supplementary Table S2).

In line with earlier findings, higher PGS increased the odds of GHD across all three BMI groups. While the effect of PGS in the low BMI group was rather modest, in medium and high BMI groups the risk of GHD were monotonically increasing with the percentile of PGS. High BMI was associated with much higher risks even compared to high PGS with medium and low BMI. Thus, this analysis confirms that the contribution of BMI outweighs the contribution of genetics for low and even medium BMIs.

Given the age of UKBB participants to be 37–73 with a mean age 56.53, for majority of cases and controls in our dataset BMI is recorded years after pregnancy with or without the occurrence of GHD. It is therefore not possible to untangle whether pre-pregnancy high BMI is a risk factor for GHD, or GHD may have triggered health conditions that resulted in higher BMI later in life. To address this question, we turn to the method of Mendelian Randomization (MR) that is widely used to infer causal relationships between two complex traits. The underlying hypothesis of the MR method is that genetic variations (SNPs) can be used as instruments to test for a causal relationship between two exposures and outcomes [29].

### 3.3. GHD Risk and Female-Specific Anthropometric Measures

To identify risk factors for GHD, we ran two-sample MR analysis using the TwoSampleMR package in R. For exposure, genetic instruments for BMI, waist circumference, hip circumference, and glycaemic traits (glucose, glycated hemoglobin) from the MR-Base GWAS catalog [30] were used. For the outcome, we used the ‘non-cancer illness code, self-reported: gestational hypertension/preeclampsia’ (ukb-b-13535) from the UKBB and four gestational hypertensive disorders from the FinnGen project as presented in the MR-Base GWAS catalog [30]. These are gestational [pregnancy-induced] hypertension, preeclampsia, preeclampsia or eclampsia, and preeclampsia superimposed on chronic hypertension.

**Figure 3:**
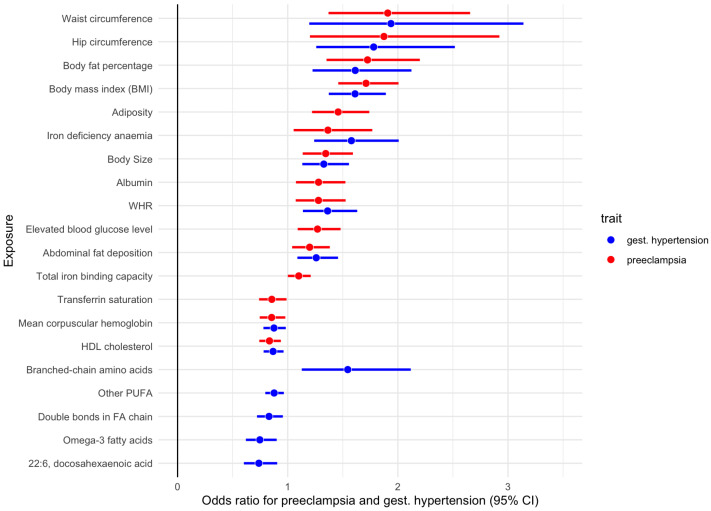
Effect of genetically proxied anthropometric measures and biomarkers on the odds of GHD. For details see Supplementary Table S5.

An earlier MR study provided evidence for causal effects of maternal systolic blood pressure and BMI on preeclampsia, eclampsia as well as low fetal birth weight [31]. Our MR analyses confirm that genetically proxied anthropometric measures such as BMI, waist circumference, and body fat percentages significantly and causatively increase the risk of GHD (Figure 3 and Supplementary Table S5). For example, the risk of preeclampsia (finn-b-O15) was greater in women with higher genetically proxied waist circumference (ieu-a-63; OR = 1.87), followed by body fat percentage (ukb-b-8909; OR = 1.72), BMI (ukb-a-248; OR = 1.69), and hip circumference (ukb-b-15590; OR = 1.44).

In the further analysis we used female-specific waist-to-hip ratio (WHR) and four anthropometric measures, specifically body size, adiposity, abdominal fat deposition, and lean mass, computed from fourteen anthropometric traits from the UKBB data through principal component analysis [32]. MR analysis identifies some anthropometric measures as causal risk factors for GHD. Specifically, female-specific adiposity that reflects an increase in fat mass at the expense of lean mass is the contributing risk factor to preeclampsia or eclampsia (OR = 1.46), Additionally, higher body size that describes a slightly disproportionate increase in body mass compared to height, resulting in higher BMI, increases the odds of preeclampsia by over 30 (OR = 1.35), followed by WHR (OR = 1.28), Interestingly, weight-neutral abdominal fat deposition is also identified as a significant risk factor for gestational hypertension (OR = 1.26), and preeclampsia (OR = 1.2).

Adiposity was reported earlier to have much stronger effects on many obesity-related diseases, including diabetes, hypertension, hypercholesterolemia, and ischemic heart disease [32]. Similarly, predisposition to abdominal fat deposition, despite being weight- and BMI-neutral, was found to be a risk factor for GHD (Figure 3 and Supplementary Table S5). Our earlier study found that female-specific WHR, adiposity, and weight-neutral abdominal fat deposition are risk factors for gestational diabetes mellitus [22] with WHR being the top risk anthropometric risk factor.

### 3.4. GHD Risk and Other Biomarkers

We further interrogated the effect of genetically proxied biomarkers on the odds of GHD. Our MR analyses confirm that genetically proxied elevated blood glucose level is significantly associated with higher odds of preeclampsia (OR = 1.24), as well as gestational hypertension, and eclampsia (Figure 3 and Supplementary Table S5). These findings are in line with a recent MR study that found the causal effect of genetically predicted diabetes with a higher risk of preeclampsia [31].

Observational studies have further suggested that preeclampsia is associated with a low intake of omega-3 long-chain polyunsaturated fatty acids (LCPUFA). Specifically, women with the lowest levels of omega-3 fatty acids were 7.6 times more likely to have had their pregnancies complicated by preeclampsia as compared with those women with the highest levels of omega-3 fatty acids [33]. Furthermore, these authors report that 15% increase in the ratio of omega-3 to omega-6 fatty acids was associated with a 46% reduction in risk of preeclampsia.

In line with these reports, our MR analyses identified that elevated levels of omega-3 fatty acids (met-c-855), and docosahexaenoic acid (met-c-852) significantly lower the odds of gestational hypertension (OR = 0.75). Adequate levels of other fatty acid metabolites, including a long chain fatty acid 10-nonadecenoate, monounsaturated omega-9 fatty acid oleic acid, and adrenate, a very long-chain fatty acid, also lower the risk of GHD. Furthermore, “other polyunsaturated fatty acids than 18:2” (met-c-917) have a similar albeit slightly weaker effect on the risk of GHD (OR = 0.88). Interestingly, the ratio of omega-6 fatty acids to omega-3 fatty acids slightly increases the odds of GHD (OR = 1.16), while the ratio of omega-3 fatty acids to total fatty acids lowers the odds of GHD (OR = 0.85), This is again consistent with the reported observation that a 15% increase in the ratio of omega-3 to omega-6 is associated with a reduction in risk of preeclampsia [33]. Consistent with these are the findings that the average number of double bonds in a fatty acid chain (met-c-851) and the ratio of bisallylic groups to total fatty acids (met-c-845) also causatively lower the odds of GHD. Fatty acid chains with double bonds are unsaturated, while those with more than one double bond are called polyunsaturated. In other words, higher levels of polyunsaturated fatty acids may play protective roles in GHD.

Another finding worth mentioning is that “good” cholesterol is negatively associated with the incidences of GHD, while “bad” cholesterol (low-density lipoprotein cholesterol levels) shows the tendency to increase the risks. For example, HDL cholesterol (ukb-d-30760) lowers the risk of preeclampsia (OR = 0.83).

It is well known that both iron deficiency, as well as iron excess, lead to adverse pregnancy outcomes resulting in a U-shaped curve. Both low and high maternal hemoglobin was found to be associated with adverse maternal outcomes, including preeclampsia [34]. Our MR analysis provides evidence that genetically proxied iron anemia increases the risk of gestational hypertension (OR = 1.58), and preeclampsia (OR = 1.36). Furthermore, genetically proxied total iron-binding capacity (TIBC) that rises when iron levels are low, has an increasing effect on the odds of preeclampsia or eclampsia. Transferrin saturation which is the ratio of serum iron and TIBC, and therefore becomes very low at iron anemia, shows anti-correlation with the incidences of GHD with marginal statistical significance (OR = 0.85). These results align with the findings on the effect of iron deficiency anemia on the risk of GHD.

Also, marginally significant associations are found between genetically proxied albumin and the risk of GHD (OR = 1.25). While high albumin to globulin ratio can occur during pregnancy, high albumin may also indicate kidney disease as reviewed in [35].

## 4. Discussion

Gestational hypertensive disorders (GHD) are the leading cause of maternal and fetal morbidity globally. They adversely affect maternal health causing long-term organ damage. Furthermore, they often lead to preterm birth and hence low birthweight that have long-term consequences for the child’s future health. Evidence suggests that the administration of low-dose aspirin initiated before 16 weeks’ gestation significantly reduces the rate of preterm preeclampsia [36]. Therefore, it is important to identify women at high risk of preeclampsia and other GHD during the first trimester of pregnancy, or before they become pregnant, thus allowing timely therapeutic interventions [1].

There is strong evidence that preeclampsia is a complex disease that involves programming changes at DNA, epigenetic, transcriptomic, protein, and metabolite levels. Clinically, the application of understanding how each of these elements affects the process of the disease state may be able to provide insights into treatment and prevention [37]. In this study, we develop and validate a polygenic risk score (PGS) for GHD, including preeclampsia, and demonstrate its high predictive performance.

While the translation of PGS for GHD into clinical practice has a number of steps [38], the development of an accurate PGS is definitely the first one. Our cross-validated model, predicts odds ratio in the high-risk group (top 1–2%) of 11.2 which implies that the odds of developing GHD in this group is more than 1100% higher than the odds of developing the condition in the low-risk group. These results did not change much if we extended the control groups to all women who gave live births, or if we narrowed the cases by removing samples with the diagnosis of gestational hypertension without proteinuria.

### 4.1. Functional Analysis

The PGS is computed based on 115 SNPs, over half of them are annotated with coding genes (Supplementary Table S3). These genes play roles in DNA repair functions (FTO, INO80, RAD51B); mitotic spindle assembly (FGD4, ABR, TOP2A, ACTN4, PREX1); transport of small molecules (FURIN, SLC39A1, FGD4, ABR, TOP2A, ACTN4, PREX1); immune system (ERBB4, FBXL18), or hemostasis (ACTN4, RAD51B) (Supplementary Table S3). Other genes participate in various metabolic processes (CDS2, GLTP, MTHFR, NDUFA10, OSBPL6, PLCE1), including glycerophospholipid biosynthesis (CDS2) and glycosphingolipid metabolism (GLTP); inositol phosphate metabolism (PLCE1); metabolism of lipids (CDS2, GLTP, OSBPL6).

In particular, it is worth mentioning the MTHFR gene that encodes the vitamin B9 (folate) dependent enzyme, methylenetetrahydrofolate reductase, which is the rate-limiting enzyme in the methyl cycle. Pregnant, and trying to conceive women, are generally recommended folic acid supplementation to ensure the proper functioning of the methylation cycle.

SNPs in the PGS score are significantly enriched in several blood pressure-related traits, including systolic blood pressure, diastolic blood pressure, pulse pressure, mean arterial pressure, interactions of blood pressure with alcohol, and smoking habits and weight-related GWAS traits (Supplementary Table S3). Specifically, the most pleiotropic SNP in the PGS, rs1421085, is mapped to the FTO gene, which is well known for its effect on obesity and anthropometric traits, type 2 diabetes, alcohol consumption, among others. The second most pleiotropic SNP (rs1458038) is mapped to the PRDM8 gene that is implicated in affecting blood pressure, atrial fibrillation, apolipoprotein B levels, LDL cholesterol levels. Another pleiotropic SNP rs167479 is mapped to the RGL3 gene that also affects several blood pressure-related traits.

Among SNPs that are not-annotated with known GWAS, the top SNP (rs1173743) significantly associated with GHD has been implicated in elevated blood pressure. It was found to be associated with lower endogenous NPR3 mRNA and protein levels in vascular smooth muscle cells, together with reduced levels in open chromatin and nuclear protein binding. The blood pressure elevating allele also increased vascular smooth muscle cell proliferation, angiotensin II-induced calcium flux, and cell contraction [39]. The second most significantly associated SNP (rs4660586) that negatively affects the risk of GHD, is mapped to the HIVEP3 gene, which regulates RUNX2 expression and its activity. According to the Neale lab GWAS, this SNP negatively contributes to basal metabolic rate, self-reported hypertension, and several anthropometric traits, including trunk fat mass, hip circumference, weight, waist circumference, and body fat percentage.

Little information is available on 5 SNPs located on the X chromosome. Two of these SNPs map onto coding genes: rs780943500 maps to the transcriptional activator ELF4 with the role in innate immunity and natural killer cell function, and rs29282 maps to the FMR1 gene that may be involved in mRNA trafficking from the nucleus to the cytoplasm and is implicated in Fragile X Syndrome and Premature Ovarian Failure. Another SNP on chromosome X (rs7052106), located between genes IL2RG and MED12, is associated with uterine fibroids and several other traits, including mean platelet volume, reticulocyte traits, and platelet distribution width, as well as total testosterone levels.

### 4.2. Risk Factors for GHD

We further demonstrate the causal effect of several anthropometric measures on the risk of GHD which is in line with earlier reports. Specifically, our analysis demonstrates a significant contribution of BMI to the risk of GHD. Furthermore, we identify female-specific adiposity and abdominal fat deposition, which despite being weight- and BMI-neutral, as risk factors for GHD that are complimentary to the BMI.

Furthermore, our studies confirm that elevated glucose levels, iron anemia, cholesterol, and albumin biomarker are risk factors for GHD. We provide evidence on the importance of adequate intake of long-chain polyunsaturated fatty acids (LCPUFA) to reduce the odds of GHD. Several trials were conducted to study this effect but they returned conflicting results. A recent systematic review of the evidence identified limitations of these preeclampsia trials associated with heterogeneity and other issues [40]. Specifically, some of these trials offered high doses of LCPUFA mid-term or later. This may actually promote the disorder instead of keeping it at bay. The usage of “nature-randomized” trials (aka Mendelian randomization) enables the integration of genetics from multiple sources to identify the importance of fatty acids for the prevention, or reducing the risk, of GHD.

### 4.3. Limitations

Our study has a number of limitations.

Firstly, the overall AUC of the PGS model has a moderate value of 0.64. Nevertheless, women in the top 5% of PGS have more than the 6-fold increased risk of GHD compared to lower 50% of the score, while women in the top 2% of PGS have a more than the 9-fold increased risk of GHD. Therefore, the PGS can be used as a predictive biomarker [41] in clinical practice as it identifies a high-risk subgroup of women.

The major limitation of the PGS model is the fact that it was developed based on DNA data from the UKBB which largely includes individuals of European ancestry. Unfortunately, the shortage of genomic research data from persons of non-European ancestry is a huge problem that reduces the accuracy of PGS predictions in clinical care. Future studies in non-European populations are needed to validate PGS across populations.

Another limitation is due to the linear character of the MR analysis. On one hand, MR provides a road map for lifestyle recommendations for women to mitigate the risks of GHD suggesting that plenty of omega-3 fatty acids (starting preconception), and healthy iron levels are recommended. On the other hand, MR does not allow for the investigation of non-linear relationships between risk factors (e.g., iron) and outcomes. Hence, the obtained result that genetically proxied higher hemoglobin levels reduce the risk of GHD contradicts findings that high maternal hemoglobin concentration in the first trimester is a risk factor for pregnancy-induced hypertension [42].

## 5. Conclusions

In this study, we develop a DNA-based polygenic risk score for GHD including preeclampsia. According to a recent report, a single genetic test per individual with automated processing and bioinformatics would cost around US$ 35 [38]. While the test itself is relatively inexpensive, there are other costs associated with the deployment of PGS at a scale that may require training and educational resources. Still, compared to other available tools, the saliva-based genetic test is a low-cost early screening tool that can be used in a clinical practice, or even at home, to identify women at high risk for GHD even before they become pregnant. This would provide a window of opportunity to offer these women preventative preconception lifestyle strategies that include intake of healthy omega-3 fatty acids, maintaining healthy iron levels, and lowering risky anthropomentric measures (BMI, adiposity, and abdominal fat deposition). Furthermore, the DNA-based test would provide a stratification tool for better triage women into established screening programs, and close monitoring by healthcare providers before or early in pregnancy. The full risk model for GHD would incorporate other risk factors, such as age, anthropometric measurements, biomarkers, medical history, and genetics.

## Figures and Tables

**Table 1:** SNPs and Weights in the Polygenic Risk Score Model ^1^.

Term	Estimate	std.error	Statistic	*p*-Value	Overlapped Gene	Nearest Upstream Gene	Nearest Downstream Gene
rs1173743	0.1453	0.031	4.712	$2.45\times 10^{-6}$	NPR3		
rs4660586	−0.1570	0.034	−4.603	$4.17\times 10^{-6}$	HIVEP3		
rs77979097	0.3020	0.071	4.266	$1.99\times 10^{-5}$	CSMD1		
rs29282	0.2175	0.053	4.128	$3.66\times 10^{-5}$	FMR1		
rs144401118	0.5282	0.130	4.068	$4.75\times 10^{-5}$		RP11-102N11.1	SMLR1
rs167479	−0.1247	0.031	−4.000	0.0001	RGL3		
rs193168008	1.1161	0.282	3.965	0.0001		LINC01019	IRX1
rs534036441	0.9111	0.234	3.901	0.0001		RNU6-163P	LINC00704
rs537363408	1.0705	0.275	3.893	0.0001	RAD51B		
rs190092234	1.1370	0.294	3.865	0.0001	OSBPL6		
rs141667164	1.2467	0.326	3.830	0.0001		DEF6	PPARD
rs115654387	0.4269	0.112	3.811	0.0001		RP11-329N22.1	RP11-334L9.1
rs544149038	1.3523	0.360	3.753	0.0002		C14orf177	AL132796.1
rs113046103	0.2230	0.060	3.703	0.0002	SHANK2		
rs561028558	1.1228	0.303	3.700	0.0002	TOP2A		
rs191614564	0.4953	0.137	3.607	0.0003		SCRT2	SLC52A3
rs72674615	0.6124	0.170	3.604	0.0003	LINC00609; PTCSC3		
rs113882455	0.4979	0.143	3.474	0.0005		U40455.1	RPL7L1P11
rs28558138	0.1080	0.031	3.452	0.0006		TBC1D19	STIM2
rs71519836	0.2241	0.065	3.447	0.0006		MYOM2	AC133633.2
rs80043362	0.1698	0.050	3.413	0.0006	JPH2		
rs183374245	0.4377	0.129	3.400	0.0007	CACNA2D1		
rs2409532	−0.0991	0.030	−3.342	0.0008	AP000330.8		
rs145385264	0.2780	0.083	3.333	0.0009	RP11-344P13.4		
rs558954655	0.7364	0.222	3.311	0.0009	SPON2		

^1^ Table with top most significant SNPs from the polygenic risk score as the result of the machine-learning procedure. SNPs are annotated using SNPnexus. Full Table with all 115 SNPs and their weights is available in the Supplementary Table S1.

## Data Availability

The results presented in this study are available in Supplementary Material. Restrictions apply to the availability of analyzed data sets; UK Biobank data is available after completing an application procedure.

## Supplementary Materials

The following supporting information can be downloaded at: https://www.mdpi.com/article/10.3390/jpm12111826/s1, Table S1: Trained linear models for computing PGS for GHD; Table S2: Odds ratios (ORs) for GHD; Table S3: Annotations of SNPs from PGS; Table S4: Trained Linear Models for computing PGS for Preeclampsia; Table S5: Mendelian Randomisation results for GHD.
